# Dual-energy spectral detector computed tomography differential diagnosis of adrenal adenoma and pheochromocytoma: Changes in the energy level curve, a phenomenon caused by lipid components?

**DOI:** 10.3389/fendo.2022.998154

**Published:** 2023-01-06

**Authors:** Yu-li Wang, Xiao-lei Liu, Ze-bing Liao, Xiao-mei Lu, Ling-lin Chen, Yi Lei, Han-wen Zhang, Fan Lin

**Affiliations:** ^1^ Department of Radiology, the First Affiliated Hospital of Shenzhen University, Health Science Center, Shenzhen Second People’s Hospital, Shenzhen, China; ^2^ CT Clinical Science, Philips Healthcare, Shenyang, China

**Keywords:** dual-energy spectral detector computed tomography, adrenal adenoma, pheochromocytoma, lipid, virtual monoenergetic image, iodine concentration

## Abstract

**Background and objectives:**

Pheochromocytoma and adrenal adenoma are common space-occupying lesions of the adrenal gland, and incorrect surgery may lead to adrenal crisis. We used a new method, dual-energy spectral detector computed tomography (SDCT), to differentiate between the two.

**Materials and methods:**

We analysed the imaging images of patients with SDCT scans and pathologically confirmed adrenal adenomas (n=70) and pheochromocytomas (n=15). The 40, 70, and 100 KeV virtual monoenergetic images (VMIs) were reconstructed based on the SCDT arterial phase, and the correlation between the arterial/venous phase iodine concentration (AP-IC/VP-IC), the effective atomic number (Z-effect), the slope of the Hounsfield unit attenuation plot (VMI slope) and the pathological results was tested. The Shapiro−Wilk test was used to determine whether the above data conformed to a normal distribution. For parameters with P greater than 0.05, Student’s t test was used, and the Mann−Whitney test was used for the remaining parameters. A ROC curve was drawn based on the results.

**Results:**

Student’s t test showed that the 40 KeV VMI and the VMI slope were both statistically significant (P<0.01). The Mann−Whitney U test showed that ID-A was statistically significant (P=0.004). ROC curve analysis showed that 40 keV VMI (AUC=0.818), AP-IC (AUC=0.736), difference (AUC=0.817) and VMI-Slope (0.817) could be used to differentiate adrenal adenoma from pheochromocytoma.

**Conclusion:**

The effect of lipid components on SDCT parameters can be used to differentiate adrenal adenoma from pheochromocytoma.

## Introduction

The adrenal gland, as an important organ of human endocrine regulation, plays an important role in the regulation of hormones in the human body. According to the updated version of adrenal lesions in the WHO 2017, different types or different growth sites of adrenal lesions can lead to different clinical symptoms (1). Pheochromocytoma originating from the sympathetic nerve can cause patients to have significantly unstable blood pressure changes through the secretion of catecholamines (2). According to previous literature reports, there are also different types of adrenal masses growing on the same side/different side of a single patient and even the occurrence of collision tumours (3, 4).

Adrenal lesions are usually difficult to distinguish on imaging and need to be identified in combination with clinical examination indicators (5, 6). Some studies even show that CT may mislead endocrinologists in the diagnosis of adenomas and propose that adrenal vein sampling is the gold standard for the diagnosis of adrenal lesions (7). In some of the latest imaging studies of adenomas and pheochromocytomas, radiologists have even put forward a very popular research word - “lipid-poor adenomas”, which have the same clinical manifestations as conventional adenomas (8). However, there is insufficient evidence for the existence of such tumours, either pathologically or clinically. Usually, these tumours have the same cellular components as conventional adenomas (Cushing adenoma, Conn adenoma, nonfunctioning adenoma) pathologically—mature fat in the cytoplasm (9). However, it is undeniable that the key to distinguishing conventional adenomas or lipid-poor adenomas from other adrenal lesions is based on whether they contain lipids.

Compared with adrenal adenomas, the highest incidence of adrenal incidentalomas (AIs) (75%-80%) and misdiagnosed patients with pheochromocytoma (0.3%-5.1%) were mistakenly resected, resulting in the adrenergic storm and life-threatening haemodynamic crisis being undoubtedly more deadly (10). In this regard, the basis of many clinical imaging studies is mainly on the morphological differences between the two (11). In general, computed tomography (CT) is better than magnetic resonance imaging (MRI) for lesion assessment. This kind of examination not only overcomes the influence of respiratory motion artefacts and obtains high-quality images but also has the advantages of a fast scanning speed and a low price (12, 13). On the basis of conventional CT, dual-energy spectral detector CT (SDCT) provides more parameters that can be used to evaluate lesions, which can be used for better evaluation of adrenal lesions. In particular, the key component that distinguishes adrenal adenomas from pheochromocytoma is lipid-containing components because pheochromocytoma often has necrosis and haemorrhage and rarely contains lipid-containing components (14, 15). At present, SDCT is widely used in the assessment of digestive, urinary and cardiovascular diseases (16–18). Compared with conventional CT to delineate the area of interest (ROI) and measure the Hounsfield unit (HU) value, SDCT can evaluate whether there is lipid in the lesion through virtual monoenergetic images (VMIs) and plotting Hounsfield unit attenuation plots (19). There is no report on the application of this technology to adrenal lesion research.

This study retrospectively analysed patients with SDCT scans and pathologically confirmed pheochromocytoma and adrenal adenomas to explore the potential of SDCT in adrenal diseases.

## Materials and methods

### Patient information

From January 2020 to May 2022, 157 patients with adrenal lesions scanned by SDCT (IQon Spectral CT; Philips Healthcare, the Netherlands) in our centre were enrolled. The inclusion criteria were: (1) Preoperative adrenal SDCT enhanced scan, completed SDCT image data, and surgical treatment within 1 month after examination; (2) Adrenal adenoma and pheochromocytoma diagnosed by pathology after the patient had surgical treatment. The exclusion criteria were: (1) CT image quality that did not meet the evaluation requirements (such as titanium clip artefact, etc. (2) The lesion was too small to affect the selection of the ROI. (3) Patients with adrenal mass recurrence undergoing secondary surgery. (4) Patients with different types of lesions or collision tumours in the ipsilateral adrenal gland. Finally, 85 patients were included, including 44 males and 41 females. There were seventy patients with adrenal adenoma, aged 23-82 (49 ± 12) years; there were 15 patients with adrenal pheochromocytoma, aged 34-75 (53 ± 13) years. This study was approved by the local ethics committee.

### Dual-layer spectral detector computed tomography scanning solution

SDCT was used for scanning. Before the CT examination, the patients fasted for 4 to 6 hours prior to intramuscular injection of antispasmodic drugs or oral contrast agents. The scan range was from the top of the diaphragm to the level of the lower edge of left kidney, covering the whole lesion. The scanning parameters used were as follows: collimator width 64 × 0.625 mm, tube voltage 120 kVp, automatic tube current control (78–145 mA), radiograph tube rotation speed 0.5 s/cycle, pitch 0.969. reconstruction layer thickness 1.0 mm, and layer spacing 0.5 mm. The enhanced scan was performed with contrast agent intelligent tracking threshold trigger technology. The trigger point was set in the abdominal aortic lumen at the celiac trunk, and the trigger threshold was 120 Hounsfield units (HUs). The venous phase started 30 seconds after the end of the arterial phase scan. Iopramide (350 mg/mL iodine concentration) was used as the contrast agent at a dose of 1.2 mL/kg, and the injection rate was 3.0 mL/s.

### Image analysis methods

VMI was reconstructed based on CT arterial phase images using a Philips SpDS imaging workstation (Spectral Diagnostic Suite 6.5). In the range of 40-100 keV, the VMI was reconstructed every 30 keV to obtain 40, 70, and 100 keV VMI maps. At the same time, the IC images of the arterial and venous phases (AP-IC and VP-IC) and Z-effect maps were obtained through the postprocessing workstation, for a total of 6 sets of images. Two senior radiologists used a blinded method to jointly evaluate the tumour area and divide the tumour area: the largest slice of the tumour was selected, and a circular ROI was drawn in this area to delineate the entire tumour, avoiding the tumour edge, other tissues or organs, and the surrounding inflammatory reaction area. The ROI was 30-5000 mm^2^, and the size and position of the ROI were not changed. The main imaging manifestations of the lesions were divided into solid, solid-cystic and cystic. By adjusting different KeV values, the average HU value of the corresponding parts was measured in the three groups of VMI. The average Z-effect value and the AP-IC and VP-IC values of the corresponding parts were also obtained. The slope of the Hounsfield unit attenuation plot was calculated as: (VMI-Slope) = (40 KeV HU-100 KeV HU)/60 KeV.

### Pathological analysis method

The resected specimens of the adrenal lesions were fixed in 10% neutral formalin, dehydrated, embedded, sectioned, and deparaffinized. Routine HE and immunohistochemical staining (MaxVision method, detection of human monoclonal antibody D2-40, Calretinin, HBME-1, CK, Vimentin and MaxVision) were performed.

### Statistical analysis

We used the Statistical Package for the Social Sciences (SPSS v. 19, Chicago, Illinois) for data statistics. The intra/interclass correlation coefficients (ICCs) were used to calculate the consistency between reviewers. A two-sided p value <0.05 was considered statistically significant. The Shapiro−Wilk test was used to test whether the data met the normal distribution. Normally distributed data were tested using Student’s t test, and we used the Mann−Whitney test for nonnormally distributed data. Various imaging measurement parameters were compared and tested. After correction using the Bonferroni test, P<0.01 indicated statistical significance. ROC curves were drawn according to the above statistical results. All statistical graphs were produced using GraphPad Prism 7 (Prism 7, La Jolla, California).

## Result

**Figure 1:**
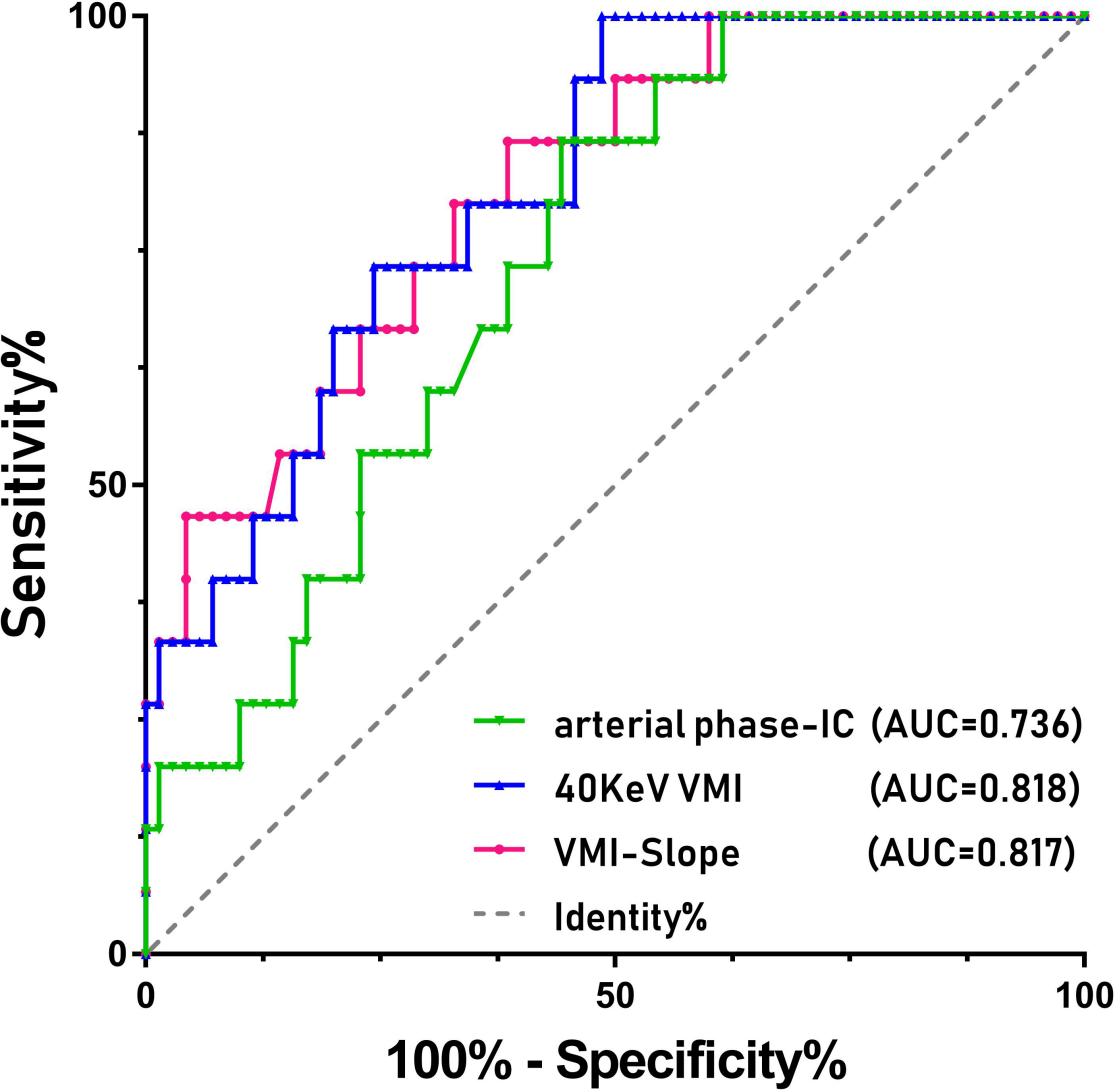
ROC curves of the various parameters of SDCT for differentiating between pheochromocytoma and adrenal adenoma in patients with adrenal lesions.

The patient’s clinical characteristics, imaging characteristics and pathological results are organized as shown in **Table 1**. The interobserver agreement of all measurements between the two reviewers was excellent, with ICC values greater than 0.80. Student’s t test results showed that the 40 KeV VMI and VMI-Slope were statistically significant (P<0.01). The results of the Mann−Whitney U test showed that the 70 keV VMI (P=0.007), the 100 keV VMI (P=0.008) and the AP-IC (P=0.004) were statistically significant in differentiating adrenal adenoma from pheochromocytoma. The ROC curve results showed that the 40 KeV VMI (AUC=0.818, CI: 0.714-0.922), VMI-Slope (AUC=0.817, CI: 0.707-0.927), 70 KeV VMI (AUC=0.724, CI: 0.617-0.855), 100 KeV VMI (AUC=0.718, CI: 0.584-0.841) and AP-IC (AUC=0.736, CI: 0.617-0.855) were statistically significant (**Figure 1**).

**Table 1:** Clinical characteristics, imaging characteristics, pathological results and SDCT parameters based on differentiating between pheochromocytoma and adrenal adenoma.

Variable	Pathological Results		*p*
	adrenal adenoma (n=70)	pheochromocytoma (n=15)	
Clinical characteristics			
Age, years*	48.74 ± 11.54	53.07 ± 13.45	0.262
Sex※			0.206
Male	34(48.6%)	10(66.7%)	
Female	36(51.4%)	5(33.3%)	
CT imaging characteristics			
Lesion size (mm^2^)	185.86 ± 91.46	2749.27 ± 1232.98	<0.001
Tumour components (cystic/solid-cystic/solid)	64/6/0 (91.4%/8.6%/0)	4/3/8 (26.7%/20.0%/53.3%)	
SDCT parameters			
40 keV VMI*	126.41 ± 76.26	229.04 ± 80.97	<0.001
70 keV VMI※ 100 keV VMI※ VMI-Slope*	60.05 ± 64.25 42.20 ± 33.20 1.46 ± 0.95	86.00 ± 50.00 57.00 ± 15.00 2.86 ± 1.17	0.007 0.008 <0.001
AP-IC※ VP-IC※ Z-effect*	1.41 ± 1.59 1.56 ± 1.01 8.08 ± 0.59	2.27 ± 1.23 2.47 ± 2.20 8.38 ± 0.53	0.004 0.207 0.060

Ps: p<0.01 indicates statistical significance. (* Data conform to a normal distribution, and values are the mean ± SD. ※Data did not conform to a normal distribution, and values represent the median ± interquartile range (IQR).

## Discussion

SDCT has been applied to the study of adrenal lesions, and several studies have shown that the use of SDCT to differentiate adrenal adenomas from adrenal metastases or adrenal glands can be very good (20, 21). In this study, the parameters of SDCT can be better used for the differential diagnosis of pheochromocytoma and adrenal adenoma. The results suggest that the lower the energy level of VMI, the better the performance in the differential diagnosis of adrenal adenoma and pheochromocytoma. Theoretically speaking, the original data space has undergone anti-correlation noise suppression and can basically achieve constant low noise across the full energy spectrum. A study in 2019 showed that the 72 KeV VMI imaging level is basically the same as the traditional CT imaging level (22). At the same time, compared with high-level VMI, because the lower single energy level of 40 KeV and 50 KeV is infinitely close to the 33 KeV level of the iodine K-edge while ensuring a lower noise level, the soft tissue contrast is improved, and the lesions are easier to detect (23). This study found that compared with the higher energy levels of 70 KeV and 100 KeV, the low energy level of 40 KeV can be better used in the differential diagnosis of adrenal adenoma and pheochromocytoma. At the same time, the VMI-Slope can also be used to distinguish the two, which may be caused by lipids in adrenal adenoma cells. In our study, AP-IC was also used to differentiate adrenal adenomas from pheochromocytomas. The Z-effect is the effective atomic number map of the material by using the X-ray attenuation degree to have the same attenuation effect on the material as a certain element. However, this study focused on the overall outline of the lesion and analysed whether the two internal substances would affect the performance of SDCT, which made the internal substances more mixed, and it was difficult to use the Z-effect for differential diagnosis. This technology can still better prompt doctors to find lesions.

The differential diagnosis of adrenal tumours and pheochromocytoma has always been one of the hot research topics in adrenal disease. In recent years, a number of studies based on traditional CT have been conducted to determine the differential analysis between the two. For example, Kang et al. developed a new evaluation system based on traditional CT to distinguish adrenal adenomas from pheochromocytoma, including attenuation of HU value, wash-out, cystic degeneration in the lesions, etc. (24). Marty et al. used artificial intelligence analysis to determine the safety thresholds for the above parameters on conventional imaging (25). There are even studies that directly use the maximum meridian and HU value of the tumour to directly classify adenomas and pheochromocytomas (26). However, these studies place too much emphasis on the differential diagnosis of wash-out. In their study, Akbulut et al. proposed that adenomas and pheochromocytomas may have similar imaging and wash-out features while ignoring an important point for their differentiation—the potential lipid-containing components within the adenoma (27).

**Figure 2:**
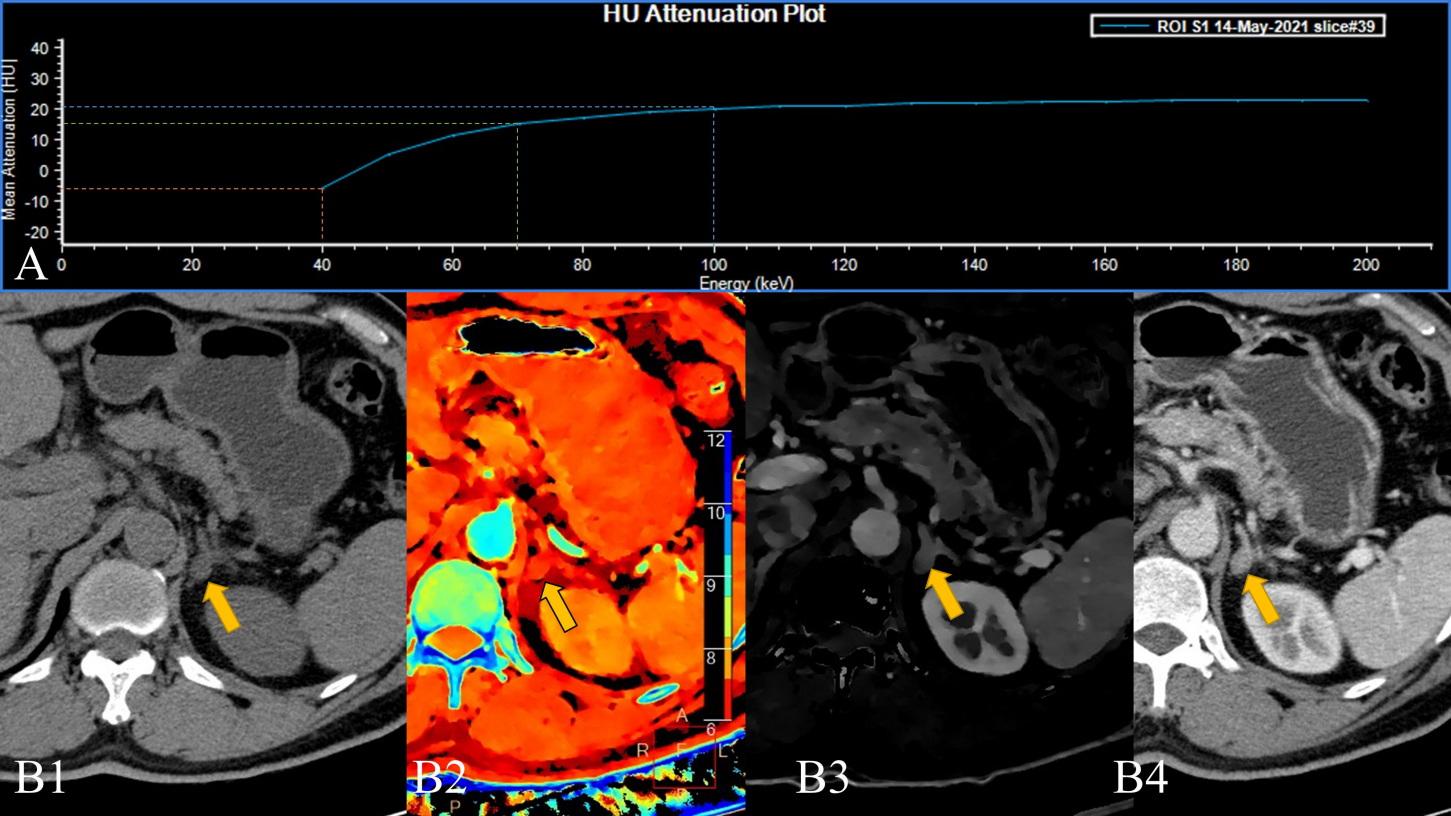
A 58-year-old male patient with a left adrenal lesion (yellow arrow); the pathological result was adrenal adenoma. **(A)**. The 40–200 keV HU attenuation plot (revealing an “inverted” curve). **(B1)**. Routine plain scan, **(B2)**. The effective atomic number can measure the effective atomic number of the lesion, and **(B3)**. Arterial phase iodine concentration (AP-IC) has poor resolution, but it can quantitatively analyse the iodine concentration in the lesion. **(B4)**. The 40 keV VMI based on arterial phase-enhanced reconstruction can not only perform a more detailed analysis of the lesion but also improve the contrast.

Obviously, the low-level VMI of SDCT has a more refined effect on the scanned object than the high-level VMI, which is similar to an amplification effect (16). At the same time, the slope of the Hounsfield unit attenuation plot (VMI-Slope) plotted in combination with high and low energy levels is even an extreme case where the curve is inverted (**Figure 2**). The reason for this is the lipids within the lesions. The effect of lipids is weak at higher energy levels.

**Figure 3:**
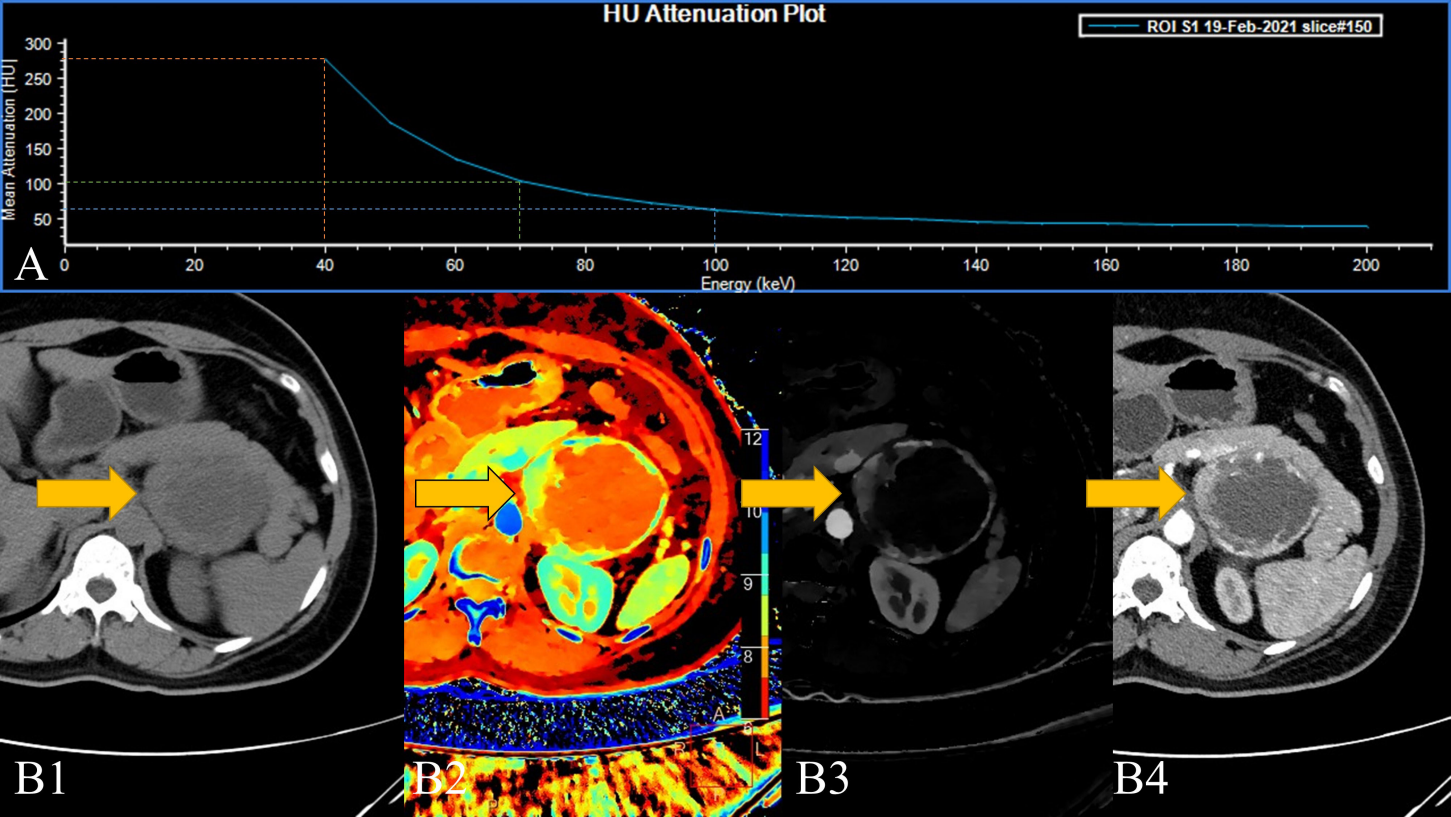
A 45-year-old female patient with a left adrenal lesion (yellow arrow); the pathological result was pheochromocytoma. **(A)**. The 40–200 keV HU attenuation plot. **(B1)**. Routine plain scan, **(B2)**. The effective atomic number can measure the effective atomic number of the lesion and help detect the lesion, and **(B3)**. AP-IC. **(B4)**. The 40 keV VMI based on arterial phase-enhanced reconstruction.

In some of the adrenal adenomas we collected, the higher VMI (70/100 KeV) lesions were positive in the overall measurement of the lesions, while the measurement value at 40 KeV turned into a negative value. This results in a Hounsfield unit attenuation plot that is the exact opposite of that seen in pheochromocytoma (**Figure 3**). From an imaging point of view, pheochromocytomas are prone to necrosis, the conventional CT measurements of liquefied-necrotic areas are usually between -5 and 15 HU, and the HU value of lipids in adrenal adenomas will be lower than this (28). At the same time, after the “amplification” effect of SDCT low-level VMI, this phenomenon is more obvious. Although in most of the measurements of adrenal tumours in our experiments, an inverted energy level curve was not exactly produced. However, as a result of statistical analysis, intralesional fat significantly lowers the 40 KeV VMI measurement, resulting in a reduced slope, flattening, and even “inversion”. Therefore, 40 keV VMI and VMI-Slope have the potential to differentiate adrenal adenoma and pheochromocytoma.

In this study, AP-IC was also found to be statistically significant in differentiating adrenal adenoma from pheochromocytoma. Although our conclusion is consistent with the consensus of these two lesions on traditional imaging, the enhancement degree of pheochromocytoma in the arterial phase is significantly stronger than that of adrenal adenoma, so its iodine concentration in the arterial phase is significantly higher than that of adrenal gland tumours (29). However, since the lesions delineated in this study contain internal cystic degeneration, necrosis, and fat components and since these components themselves are not enhanced, the area of these areas directly affects the overall iodine uptake rate of the lesions. In our study, we cannot directly believe that VP-IC, which is not statistically significant, cannot be used to differentiate between adrenal adenoma and pheochromocytoma, but it is certain that the efficacy of distinguishing the two in the arterial phase is obviously better.

Furthermore, in some pioneering studies, scholars have been able to identify the adrenal tumour parenchyma and cystic necrosis components by using positron emission tomography (PET)-CT and dynamic contrast enhanced (DCE) techniques. In pheochromocytoma, especially that presenting with cystic components, the active areas of tumour metabolism are concentrated in the relatively thin cyst wall of the tumour (30). This finding can effectively be used to differentiate pheochromocytoma from other benign cystic masses. Although SDCT can provide more refined quantitative parameters, such as VMI-Slope for distinguishing lipid containing components, it seems that at present, it is still unable to effectively distinguish the metabolically active region of the tumour, and it cannot be refined to ascertain the imaging changes caused by the pathological components (31). Further improvements in CT technology or the application of image-pathology omics analysis could potentially help draw more definitive conclusions in the future.

In addition to the abovementioned limitations of lesion delineation, the limitations of this study include the following points: 1. Since this study only retrospectively analysed data for one and a half years, the sample size was small, and the distribution of the two groups of samples was uneven. The sample size of adrenal adenomas was significantly higher than that of pheochromocytomas due to incidence. We will continue to collect samples to verify the findings of our research. 2. The conclusions of this study obviously cannot be directly applied to clinical practice. Because adrenal lesions are obviously not only adenomas with fatty components, adrenal myelolipoma is one of these lesions (32), and they require more analysis and the identification of more different types of diseases. 3. Most adrenal lesions are relatively small, and it is difficult to delineate them. If more advanced technology can be used for image segmentation and extraction of lesions, the accuracy of the study will be further increased. 4. Although we identified the main components of the lesions in this study, the impact of each component on the SDCT parameters is unknown, and further research is needed to confirm our findings. However, this study still draws an interesting conclusion that the SDCT energy spectrum curve is a technique with potential analytical power in adrenal lesions, especially for the identification of lipid-containing tissue or lesions.

In conclusion, the parameters of SDCT, low-level VMI, VMI-Slope, and IC can be used to differentiate adrenal adenomas from pheochromocytoma. The slope of the Hounsfield unit attenuation plot has potential application for lipid-containing lesions.

## Data availability statement

The original contributions presented in the study are included in the article/supplementary material. Further inquiries can be directed to the corresponding authors.

## Ethics statement

The studies involving human participants were reviewed and approved by Shenzhen Second People’s Hospital Ethics Committee. The patients/participants provided their written informed consent to participate in this study.Written informed consent was obtained from the individual(s) for the publication of any potentially identifiable images or data included in this article.

## Author contributions

YL-W contributed to the conception of the study; HW-Z, XM-L and LL-C contributed significantly to analysis and manuscript preparation; LF performed the data analyses and wrote the manuscript; Z-B L helped perform the analysis with constructive discussions.
